# Enablers and barriers of General Practitioner’s choice of additional skills training: a mixed-methods study

**DOI:** 10.3389/fmed.2025.1506396

**Published:** 2025-05-26

**Authors:** Abdul-Aziz Seidu, Emma M. Anderson, Hannah Mason, Francis A. Albert, Faith O. Alele, Paula Heggarty, Aaron Hollins, Tarun Sen Gupta, Lawrie McArthur, Richard Hays, Bunmi S. Malau-Aduli

**Affiliations:** ^1^College of Medicine and Dentistry, James Cook University, Townsville, QLD, Australia; ^2^School of Health, University of the Sunshine Coast, Sunshine Coast, QLD, Australia; ^3^Discipline of General Practice, The University of Adelaide, Adelaide, SA, Australia; ^4^School of Medicine and Public Health, College of Health, Medicine and Wellbeing, The University of Newcastle, Callaghan, NSW, Australia

**Keywords:** advanced skills training, Australia, General Practice, generalist, rural, remote

## Abstract

**Introduction:**

Additional skills training (AST) is a prerequisite for rural generalist training in Australia, where Rural Generalists (RGs) undergo specialized training in a distinct discipline for a period of 12 months. This study investigated the perspectives of General Practitioners (GPs) regarding the factors influencing their selection of AST programs.

**Methods:**

Using a sequential explanatory mixed methods approach, quantitative survey data and qualitative interview data were collected. Quantitative data was analyzed using frequencies, percentages, mean and standard deviations, while thematic analysis was employed for the qualitative data.

**Results:**

A total of 106 respondents completed the survey, with 13 participating in interviews (supervisors *n* = 5; registrars *n* = 8). GPs perceived AST as beneficial in improving patient outcomes (57.5%) and enhancing patient satisfaction (49.1%). Intrinsic motivations for pursuing AST included personal interest, professional growth, and a desire to enhance patient care. However, funding challenges, burnout, and workload were identified as significant barriers to acquiring additional skills. Qualitative analysis identified six themes, three each related to facilitators (desire to work rurally, meeting workforce needs, and support networks) and barriers (work-life balance, mismatched expectations, and inadequate recognition of AST).

**Conclusion:**

Additional skills training is a highly valued training program. Most of the GPs who were involved in the program were intrinsically motivated to participate. However, to ensure its sustainability, wider recognition of the value, better visibility, and better alignment with community needs are required.

## Introduction

The Australian General Practice training model is a well-developed program focused on practical, in-practice teaching. Registrars gain experience in various relevant settings, training in an apprenticeship-style model where they see patients under graded supervision while contributing to the workforce (1). The services delivered by General Practitioners (GPs) including procedural services remains a major component of health delivery, especially for those living in rural and remote areas (2). Beyond procedural skills, GPs in rural areas increasingly utilize non-procedural skills, such as mental health and chronic disease management, to address local health needs (3). People living in rural and remote settings in Australia face significant challenges in accessing healthcare and have poorer health outcomes compared to their metropolitan counterparts (4–6). To address these disparities, initiatives have been introduced to enhance access to health and general practice, with rural communities depending on Rural Generalists (RGs) for comprehensive medical services (7).

A rural generalist, as defined by the National Rural Generalist Taskforce (8), is a medical practitioner trained and located in rural areas to ensure that the current and future healthcare needs of the communities they serve are sustainably and cost-effectively addressed. This includes providing general practice, emergency care, and necessary specialist components in hospital and community settings (8). In Australia, formal training for rural practice typically spans a minimum of 4 years (1). Advanced Specialized Training is provided by the Australian College of Rural and Remote Medicine to improve GPs’ knowledge in either procedural or non-procedural skills, with a minimum duration of 12 months (9). Relatedly, the Royal Australian College of General Practitioners (RACGP) also offers 12 months of Additional Rural Skills training, equipping GPs with specialized skills to meet the needs of their communities (10). In this paper, Advanced Specialist Training and Additional Rural Skills Training are referred to as Additional Skills Training (AST). AST is a requirement for GPs in Australia, aiming to improve healthcare delivery in rural and remote areas (11). Posts funded by the Program are required to take place in rural, remote, or Modified Monash (MM) 2–7 areas, unless those areas do not offer the required training (12). AST includes specialties such as Aboriginal and Torres Strait Islander health, academic post, adult internal medicine, anesthesia, child health, emergency medicine, mental health, obstetrics, palliative care, surgery, and small town rural general practice (1). It enables GPs to expand their skills beyond traditional general practice training (8).

Currently, AST is supported through a collaborative approach involving the Australian Government Department of Health and Aged Care, ACCRM, RACGP, state and Northern Territory Health Departments, National Rural Generalist Pathway Coordination Units, Local health networks, public health settings, RG registrars, GP registrars, and fellowed RGs and GPs (12). The program aims to increase support for RG and GP registrars and fellowed RGs and GPs in regional, rural, and remote Australia to develop additional skills, build a strong rural training network by increasing the number of highly skilled supervisors, and ensure rural communities have access to appropriately skilled healthcare providers (11, 13). There is widespread support for mental health and Aboriginal and Torres Strait Islander Health ASTs as priority areas, reflecting the consistent demand for these skills across rural Australia (12). However, AST programs are structured and function differently globally with a common goal of improving rural and remote health (13).

Despite a growing body of evidence on AST and its impacts, research on the enablers and barriers to training from the perspective of GPs remains limited. Existing studies have primarily focused on workload, work activities, experiences of support during AST (14), outcomes of AST (15), and the establishment and advancement of AST (16). Understanding the perspectives and needs of GPs trainees in AST programs is crucial to fostering a conducive learning environment responsive to the transformative lifelong learning (7). Achieving this objective requires continuous evaluation and monitoring of training and learning experiences. This study, therefore, seeks to address the following research questions:

(1) What are the extrinsic and intrinsic factors that influence GPs’ choice of AST?
(2) What are GPs’ perceptions of the enablers and barriers to the AST program?

The findings from this study will help identify the challenges and experiences GP face during their training and clinical practice and suggest possible measures to ameliorate such problems. This study will also help identify the extrinsic and intrinsic factors influencing GPs choice of AST.

## Materials and methods

### Ethics

Ethics approval was obtained from the James Cook University (JCU) Human Research Ethics Committee (HREC H9139). Prior to the data collection, respondents were provided with the necessary information about the study in both phases. Consent was obtained from all respondents, including consent to record the interviews.

### Study design

This study adopted a sequential explanatory mixed methods approach within the pragmatic paradigm (17, 18). The first phase of the study employed a cross-sectional quantitative study design. At this stage, quantitative data was collected on the extrinsic and intrinsic factors influencing GPs choice of AST. This data was analyzed to identify issues that warrant further exploration. In the second phase, a descriptive phenomenological study design was adopted to explore GPs’ experiences of the enablers and barriers to their choice of AST program.

### Phase one: quantitative survey

A cross-sectional survey was conducted using questionnaires among GPs to answer research question one. The questionnaire was developed using Qualtrics. It comprised two sections: demographic and practice characteristics of respondents (e.g., age, gender, current practice setting and location) and questions regarding AST experiences and perspectives. Questions regarding participant perceptions of the AST program were on a 5-point Likert scale (SD = 1 to SA = 5) (e.g., The acquisition of AST is essential for providing comprehensive and specialized patient care, pursuing AST is primarily driven to better meet the needs of patients in underserved areas). The questionnaire was piloted among five experts involved in the program before the actual data collection. The survey was refined following the feedback from the experts and finalized before deployment.

General Practitioners were invited to participate in an online survey between September and October 2023. The inclusion criteria were: being a practicing GP with completion of AST in Australia, and willingness to participate in the study. Respondents were recruited via the General Practitioners and registrar’s database. Emails were sent with the Qualtrics survey link to participants. To increase participation, respondents were asked to enter a draw to win one of five $100 gift cards. The last question in the survey was used to recruit participants for the qualitative phase of the study. Additionally, to increase participation, posters were used to invite potential participants during the Rural Medicine Australia 2023 conference in Hobart, Tasmania. Finally, snowball sampling was also employed to invite participants who met the inclusion criteria.

### Quantitative data analysis

The quantitative data were analyzed using R version 4.3.0. Descriptive statistics, including frequencies and percentages, were calculated for categorical variables. For continuous variables, mean and standard deviation were used.

### Second phase: qualitative phase

The qualitative phase of the project employed descriptive phenomenology to explore the experiences of GPs within the AST program (19). Two participant groups were selected for this study through convenience and purposive sampling: Directors of Medical Services (DMS) and Fellows. Inclusion criteria mandated that participants had completed AST in Australia. DMS were invited from a list of contacts within one of the co-authors’ professional networks (AH). Fellows were invited from a pool of participants who had completed the survey in the first phase of the project and had expressed interest in participating in an interview regarding their AST experience. Participants received compensation for their time in the form of a $50 grocery store gift voucher and were entered into a draw for a chance to win a $100 department store gift voucher.

### Qualitative data collection

The interviews were semi-structured, offering a framework for the discussion while allowing participants flexibility to elaborate on their AST experiences. Examples of interview questions included: What motivated you to pursue the AST program? How has the AST program impacted the recruitment and retention of healthcare professionals in your community? How does the program address the unique challenges facing rural clinical practice? Do you feel that the AST program has improved your ability to care for rural patients? A female research assistant (HM) received guidance from a senior researcher (EA) on the prescribed protocol and interview technique, as well as the interview guide. Neither EA nor HM had prior involvement in AST and held minimal preconceived notions about this training. EA conducted the initial interview, with HM present to observe and familiarize with the process. Subsequent interviews were conducted by HM. The interviews, conducted via video conferencing, took place between 29 September 2023, and 19 October 2023 until data saturation was reached, lasting between 20 and 45 min.

### Qualitative data analysis

The recorded interviews were transcribed verbatim using Otter AI and the transcription feature in Microsoft Teams. The transcripts underwent de-identification and were imported into NVivo version 20 (Lumivero, Colorado, United States). An inductive thematic approach, following Braun and Clarke’s 6-step process (20) was adopted for data analysis. First, two research team members (HM and AS) meticulously cleaned the transcripts and familiarized themselves with the data. Second, generating initial codes was initially piloted by three authors (HM, AS, and EA). Once consistency was established, the remaining interviews were independently coded by either HM or AS. In the third stage, HM and AS categorized the various codes into themes, systematically gathering all relevant coded data extracts associated with the identified themes. In the fourth stage, the primary coders (HM and AS) met with senior researchers (EA and BMA) to review and refine the themes identified. These themes were then defined and renamed through group consensus among the research team. Finally, verbatim representative quotes from participants were selected to support each theme in the report. Lincoln and Guba’s four criteria for ensuring trustworthiness were followed (21). The consolidated criteria for reporting qualitative research (COREQ) checklist was adhered to for reporting the findings of the qualitative study (22).

### Triangulation

The principles outlined by O’Cathain et al. (23) supported the triangulation of findings in both phases of this study (23). The process included (1) analyzing and extracting threads independently from each phase, (2) connecting the threads between the first and second phases to enable a joint interpretation, and (3) formulating comprehensive conclusions and meta-inferences through the integration and interpretation of findings from both study phases.

## Results

### Quantitative phase

#### Demographic characteristics of the respondents and practice history by location

The demographic characteristics and practice history of 106 responding GPs are presented in Table 1. The average age was 39 (± 11.28 years) with an average year of working experience of 9.33 (± 9.51 years). The participants were evenly split by gender. Almost all (98.1%) the responding GPs were in Queensland. Representation was lower in remote communities, small rural towns, large rural towns, and metropolitan areas but a strong presence in medium rural towns (27%) and regional centers (25%). Predominantly, they worked in hospitals (35%) and blended practices (31%), followed by general practice (23%) and Aboriginal Community Controlled Health Services (6.6%). The majority (82%) are Australian Medical Graduates. Fellowship of the Australian College of Rural and Remote Medicine (FACRRM) was the main curriculum (45%). Furthermore, (61%) have achieved fellowship status. More than half (56%) undertook the AST, and the majority (28%) undertook anesthetics as their AST discipline.

**TABLE 1:** Demographic characteristics of the respondents and practice history by location.

Characteristic	Overall, *n* = 106
Age, mean (SD)	39.0 (11.3)
Years of working, mean (SD)	9.3 (9.5)
**Gender, *n* (%)**	
Female	53 (50)
Male	53 (50)
**State, *n* (%)**	
Queensland	104 (98.1)
Victoria	2 (1.9)
**Rurality of work location, *n* (%)**	
Remote or very remote community (MM6 or 7)	13 (12)
Small rural town (MM5)	13 (12)
Medium rural town (MM4)	29 (27)
Large rural town (MM3)	12 (11)
Regional center (MM2)	26 (25)
Metropolitan area (MM1)	10 (9.4)
**Current work, *n* (%)**	
Hospital	37 (35)
Blended	33 (31)
GP	24 (23)
ACCHS	7 (6.6)
**Graduate type, *n* (%)**	
AMG	87 (82)
IMG	19 (18)
**Curriculum, *n* (%)**	
FACRRM	48 (45)
FRACGP and FARGP or RACGP-RG	16 (15)
FRACGP	15 (14)
FRACGP and FACRRM and FARGP or RACGP-RG	13 (12)
FRACGP and FACRRM	9 (8.5)
FARGP or RACGP-RG	5 (4.7)
**Fellowship status, *n* (%)**	
Fellowed	65 (61)
Current	41 (39)
**Advance training, *n* (%)**	
AST	59 (56)
Unknown	19 (18)
ARST	17 (16)
Extended skills	9 (8.5)
ARST and AST	2 (1.9)
Skills discipline, *n* (%)*	
Anesthetics	28 (28.0)
Emergency medicine	17 (17.0)
Obstetrics and gynecology	16 (16.0)
Aboriginal and Torres Strait Islander health	8 (8.0)
Adult internal medicine	5 (5.0)
Academic practice	5 (5.0)
Mental health	4 (4.0)
Child Health/pediatrics	4 (4.0)
Surgery	3 (3.0)
Population health	3 (3.0)
Palliative care	3 (3.0)
Small town rural practice (discontinued)	2 (2.0)
Remote medicine	2 (2.0)

*Out of 100 responses for this question. ACCHS, Aboriginal Community Controlled Health Service; AMG, Australia Medical Graduate; ARST, advanced rural skills training; FACRRM, Fellowship of the Australian College of Rural and Remote Medicine; FRACGP, Fellowship of the Royal Australian College of General Practitioners; FARGP, Fellowship in Advanced Rural General Practice; GP, General Practice; IMG, International Medical Graduate; RACGP-RG, Royal Australian College of General Practitioners-Rural Generalist; SD, standard deviation.

### GPs attitudes toward additional skills training

**FIGURE 1:**
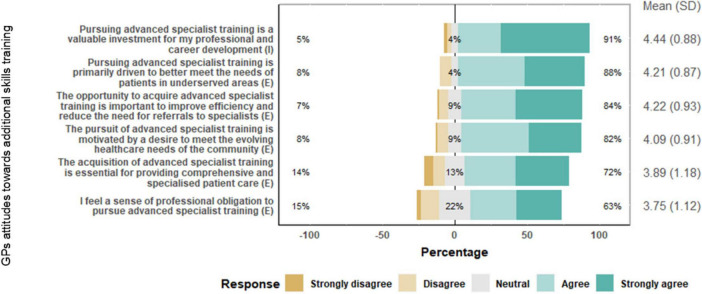
Responses to questions on commitment to excellence in healthcare as factors affecting decision to pursue additional skills training (AST).

Figure 1 presents GPs’ attitudes toward AST program. There was a high agreement about the motivators for pursuing AST. Ninety-one percent of GPs agreed that pursuing AST is a valuable investment for professional and career development, indicating a strong intrinsic motivation related to career advancement with a mean score of 4.44 out of 5. The opportunity to acquire AST to improve efficiency and reduce referrals had the second-highest mean score of 4.22. Although, still more than average, the statement with the lowest level of agreement (63%) was the sense of professional obligation to pursue AST, suggesting extrinsic motivation related to professional duty is less of a driving factor with a mean score of 3.75.

### Professional growth and community factors affecting decision to pursue AST

**FIGURE 2:**
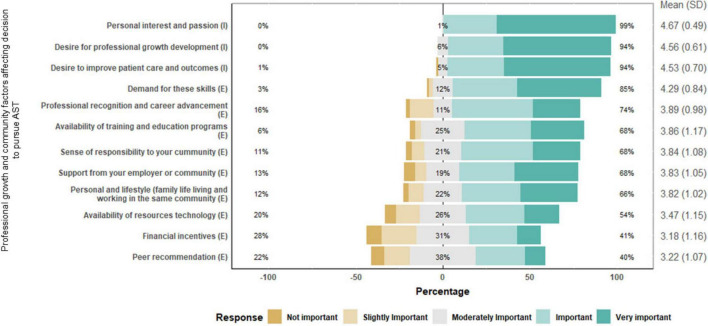
Responses to professional growth and community factors affecting decision to pursue additional skills training (AST).

In Figure 2, the responses to questions regarding professional growth and community factors affecting the decision to pursue AST were all above average. The three highest rated factors were intrinsic factors, “Personal interest and passion” (Mean = 4.67), which had a 99% importance rating, “Desire for professional growth development” (Mean = 4.56), 94% and “Desire to improve patient care and outcomes,” (Mean = 4.53), 94%. Also, 85% and 74% of the surveyed GPs rated extrinsic factors such as “Availability of training and education programs” and “Professional recognition and career development” as highly important, respectively. Other factors include “Support from your employer or community,” “Sense of responsibility to your community,” and “Demand for these skills,” which were also rated highly, with 85% and 68% of respondents, respectively, considering them important. Financial incentives, an extrinsic factor, had a relatively lower mean importance score (Mean = 3.18).

### Enablers and barriers of AST

**FIGURE 3:**
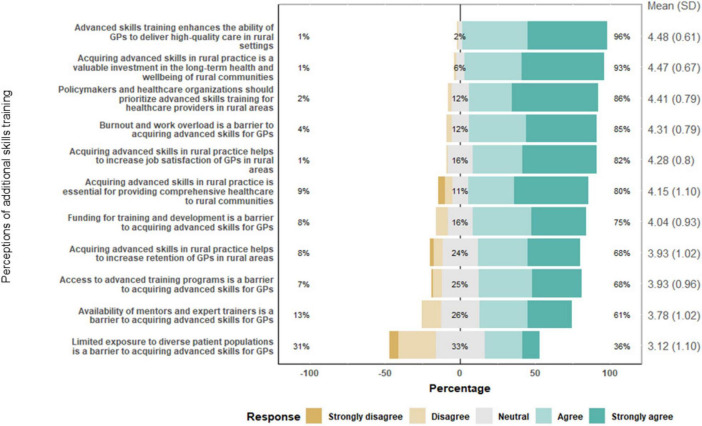
Responses to questions on enhancing skills and overcoming barriers through additional skills training (AST).

The enablers and barriers to acquiring AST among GPs are presented in Figure 3. The majority (86%) of responding GPs agreed or strongly agreed that policymakers and healthcare organizations need to prioritize AST, with a mean response of 4.41 ± 0.79. A notable divergence in opinion was observed regarding the impact of exposure to diverse patient populations on acquiring AST, with a mean score of 3.12 ± 1.10. While 36.5% agreed or strongly agreed that it was a barrier, 32.9%, remained neutral. Funding, burnout, workload, and the availability of mentors were also identified as significant barriers to AST. The mean scores for these items were 4.04 ± 0.93, 4.28 ± 0.81, and 3.78 ± 1.02, respectively, indicating general agreement with these statements. In particular, the impact of burnout and workload was acknowledged, with 85% agreeing or strongly agreeing that these factors were barriers. A high proportion of GPs agreed or strongly agreed that investment in AST was crucial for the “delivery of high-quality care in rural settings” and for “investment in the long-term health and wellbeing of rural communities,” with mean scores of 4.48 ± 0.61 and 4.47 ± 0.67, respectively, reflecting strong agreement (See Figure 3).

### Perceptions of additional skills training

**FIGURE 4:**
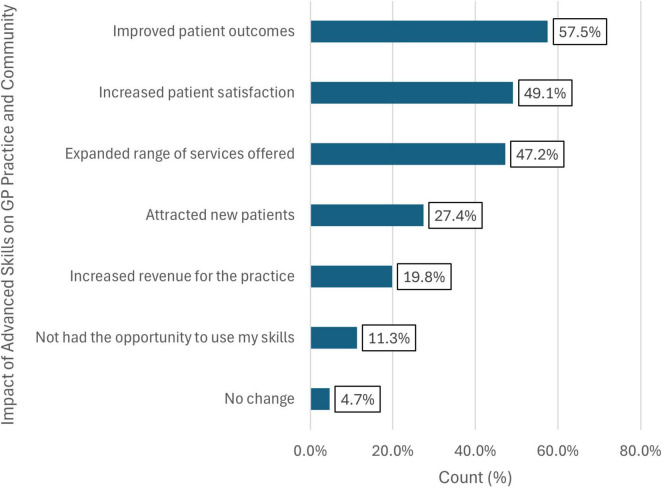
Distribution of how advanced skills have changed or impacted General Practitioners (GPs) practice or community.

The GPs were asked how AST impacted or changed their practice (Figure 4). Over half (57.5%) indicated that AST improved patient outcomes, and 49.1% reported increased patient satisfaction. Additionally, 47.2% observed an expanded range of services offered in their practice due to AST, while attracting new patients was noted by 27.4%. About 4.7% reported no change after acquiring additional skills (Figure 4).

### Additional skills discipline and frequency of utilization

As shown in Table 2, respondents were asked about the frequency of utilization of listed skillsets in their practice. The top five skills the respondents used daily were chronic disease management (60.7%), mental health (53.9%), internal medicine (52.8%), pediatric (51.7%), and adolescent and youth health (46.1%) while the least used were travel medicine (3.4%) and refugee health (1.1%).

**TABLE 2:** Frequency of skills utilization.

	Frequency of utilization (%)				
Skills	Daily	Once a week	Once a fortnight	Monthly	Once a year/rarely
Anesthetics	13.5	13.5	9.0	12.4	51.7
Aboriginal and Torres Strait Islander health	39.3	16.9	10.1	13.5	20.2
Adolescent and youth health	46.1	25.8	9.0	2.3	16.9
Academic practice or academic post	10.1	10.1	10.1	10.1	59.6
After hours medicine	14.6	34.8	12.4	7.9	30.3
Aged care	41.6	20.2	10.1	9.0	19.1
Allergy medicine	7.9	14.6	12.4	19.1	46.1
Chronic disease management	60.7	15.7	3.4	6.7	13.5
Cosmetic laser and dermatology medical	4.5	5.6	12.4	4.5	73.0
Emergency medicine	40.5	18.0	10.1	12.4	19.1
Education	33.7	23.6	13.5	9.0	20.2
Internal medicine	52.8	23.6	5.6	1.1	16.9
Mental health	53.9	21.4	6.7	5.6	12.4
Obstetrics and gynecology	29.2	28.1	13.5	10.1	19.1
Occupational medicine	7.9	11.2	21.4	15.7	43.8
Pediatrics	51.7	20.2	12.4	2.3	13.5
Palliative care	12.4	25.8	12.4	27.0	22.5
Population health	13.5	13.5	14.6	16.9	41.6
Remote medicine	25.8	7.9	10.1	15.7	40.5
Refugee health	1.1	2.3	4.5	12.4	79.8
Respiratory disease	42.7	22.5	13.5	5.6	15.7
Sexual and reproductive health	32.6	29.2	13.5	7.9	16.9
Skin cancer	18.0	27.0	10.1	15.7	29.2
Sports medicine/musculoskeletal	28.1	24.7	11.2	13.5	22.5
Surgery	13.5	12.4	15.7	16.9	41.6
Travel medicine	3.4	10.1	18.0	27.0	41.6

### Qualitative phase

Of the 13 DMS contacted, five participated in the interviews, while eight of the 17 contacted medical fellows participated. Overall, 13 participants participated in the qualitative phase, of which eight were male (61.5%) and five were female (38.5%). The most reported AST was Anesthetics (5), followed by emergency (*n* = 4), obstetrics and gynecology (*n* = 2), internal medicine (*n* = 2), and academic (*n* = 1).

Inductive thematic analysis of the data revealed six key themes concerning enablers and barriers to access and effective participation in the AST programs. The identified enablers related to factors that positively influenced their participation, while the barriers pertained to factors that negatively impacted GPs’ access to and participation in the AST program. Three of the themes (desire to work rurally–intrinsic motivation, meeting workforce needs and support networks) were related to the enablers, while the remaining three (work life balance, mismatched expectations, and inadequate knowledge and recognition of AST) were linked to the barriers.

### Enablers to the additional skills training program

#### Desire to work rurally (intrinsic motivation)

The first theme in relation to enablers of AST was the intrinsic motivation of participants to work rurally and advance their career. Some of the participants described how, while growing up in rural areas, they witnessed challenges with healthcare access, contributing to a desire to support to addressing healthcare disparities in rural and remote areas. This motivation prompted them to undertake AST to achieve this goal. Additionally, some participants expressed a lack of interest in working in metropolitan areas.

*“Well, I grew up on a farm in a rural area and we often didn’t have local doctors. And if you had to have like a simple procedure done, you would often have to travel a long way to do it. And so I’m talking like taking over 2 h to actually see doctor to have a procedure done. So I did placement as a medical student out in the [health district], in [rural town]and I really liked it.” Fellow 4, Female.*

#### Meeting workforce need

The second theme generated from the data on enablers to AST training was meeting workforce needs. Some of the fellows reported that meeting workforce needs propelled them to undertake AST. They discussed the dynamic nature of career paths in the medical field, where unexpected opportunities can lead individuals to alter their plans based on emerging possibilities and unique circumstances.

*“Originally, I had planned to do emergency medicine as an advanced skill. And I did that for, I guess the clinical experience and just getting better at emergency presentation. But then someone pulled out of the obstetric program in [regional center] and they’re like, “why don’t you apply?” So I did and I got it. So I ended up doing the obstetrics. I have always liked Women’s Health, but I didn’t particularly see myself doing rural generalist obstetrics.” Fellow 2, Female.*

#### Support networks

Another important enabler the participants discussed was the support networks available to them. Specifically, some participants emphasized the importance of organizational mentorship, professional networks, and their positive impact on their AST.

*“… I had a really great supervisor who was able to guide me, which was, really good and overall, I had a really good amount of support. I think because I wasn’t aware of other people doing an academic AST… that actually, I knew of another fellow who did it through RACGP who did an academic placement through RACGP, but I wasn’t aware.” Fellow 3, Male.*

### Barriers to the additional skills training program

Despite the positive training experiences or enablers to AST highlighted by some of the participants, some barriers were discussed. The themes generated from the data on barriers are discussed below.

#### Work-life-balance

Work life balance was a challenge that the participants felt hindered the smooth and successful participation in the AST program. In addition to that, some participants also discussed how challenges associated with rural living impacted their AST program. Some shared how they were already established and had to relocate causing a major disruption in the work and family life balance. Some of the participants also discussed the practical challenges faced by medical professionals in rural areas, emphasizing the impact on personal and professional dimensions and serving as barriers to their AST training. They discussed the interplay between housing conditions and technology infrastructure, focusing on inadequate internet coverage.

*“The biggest challenges are… the first one is… that you are already established in the community, so I was a GP and a hospital doctor in [rural town] and you’re already established in that community and you’ve literally got to pack up your life for an entire year, move away.” Fellow 3, Male.*

*“So at that point it was a boom in [Rural Town] housing was either poor quality or extremely expensive. Or very substandard. My wife was traveling with me during that time and one of the big issues was Internet coverage. We couldn’t get any decent Internet coverage.” Fellow 1, Male.*

#### Mismatch between skills and expectations

The other theme generated from the data on the barriers to AST was mismatched expectations. The participants discussed this in relation to systemic barriers and financial challenges. Most of the participants shared their experiences relating to the systemic barriers they were confronted with during their AST training. Some shared their views on the conflicting expectations from ACCRM, and the limitations imposed by their placement. The discord between these entities led to stress and challenges in meeting requirements.

*“One of the frustrations I had was that there was a very big discord between what Queensland Rural Health expected I should do, what ACCRM required me to do, and what my placement allowed me to or could afford me to do, and that caused quite a lot of angst because a concept if you don’t do this, you’ll fail. Can’t say you can’t do it. It’s like well so some of the parts I had to do in my days off rather than being so actually had to take some of the academic things off in my own time rather than doing it as a registrar under the banner of paid time.” Fellow 6, Male.*

Some participants shared their experiences on the challenges they faced during their AST training as financial issues. Specifically, some shared how their income decreased due to change in rank. Despite the financial setback, some participants felt compelled to accept the role due to high demand and competition for the position notwithstanding the financial sacrifices involved.

*“The other thing is that a lot of us, at least in my case, I was a Senior Medical Officer before I did my AST, and I was dropped to Registrar hourly rate income, and I came from an income and then it dropped by half or more. And there was no way around it because well, according to Queensland Health, you’re not supposed to change your pay scale. But on the other hand, if I don’t do it, and I don’t take the job on the pay they will have 20 more people who want to take it but just yeah, just get on board and just go in and d*o *it.” Fellow 7, Male.*

#### Inadequate knowledge and recognition of additional skills training

Most of the participants indicated how inadequate knowledge and recognition of AST is a major barrier to smooth AST program. Some discussed that specialists, particularly those in higher positions, undervalue them and shared that many specialists lack a comprehensive understanding of the work of their junior colleagues and the scope of their work. Some used the phrase “glass towers” suggesting a disconnect between those in leadership roles and the practical realities. These participants suggested that if these specialists were more involved in hands-on work, they would gain a better appreciation for the capabilities and contributions of the junior colleagues and recognize the advanced skills they have acquired.

*“I don’t think they value (us)…. they undervalue us, I think quite a lot. I think most specialists up there really don’t understand the breadth of what we actually do. I think that’s one of the biggest problems and you know, certainly the guys that do get out of the glass towers and come down and do some work, go back with much better appreciation of what we can actually achieve.” Fellow 5, Female.*

### Triangulation of study findings

Table 3 shows triangulation of the quantitative and qualitative findings.

**TABLE 3:** Triangulation of study findings.

Topic/theme	Quantitative findings	Illustrative qualitative quote	Synthesis
**Enablers**			
Desire to work rurally (intrinsic motivation)	The quantitative findings showed that most respondents are pursuing AST due to their personal interest and passion with a mean score of 4.67 ± 0.49.	*“I got sick of metropolitan practice…” Fellow 1, Male.*	This underscores the need to emphasize the importance of intrinsic motivation among GPs.
Meeting workforce needs	The quantitative data also showed that respondents were motivated to undertake AST due to the demand for those skills they trained for with a mean score of 4.28 ± 0.84.	*“I did my student placement as well as registrar years as well as going back as a fellow in the same place. So I’ve had the ability to get to know the local teams and current DMS in the point of view of saying, look, I know that this is going to be needed. I know that you don’t currently employ this type of AST. But I can bring these skills back in 2 years.” Fellow 6, Male.*	Ensuring that AST is delivered to fill specific needs is crucial. Also prolonged and consistent engagement with the same institution suggests a deep familiarity with local needs and challenges, which is important for rural settings. By developing strong local relationships and understanding specific requirements, RGs can effectively introduce new skills to meet the needs of the communities they serve.
Support networks	The quantitative results showed that support from employer or community is a major factor affecting their decision to pursue AST with a mean score of 3.83 ± 1.05.	*“I was much more able to do the emergency department work that I was required to do in the rural hospital, and I got some very clear direction and support about what courses to do.” Fellow 1, Male.*	Support networks are essential in every human endeavor. This emphasizes that having clear direction and support in training enhances RGs confidence and self-efficacy and overall ability to perform effectively. This structured guidance ensures that RGs are well-prepared and competent, leading to improved patient care and overall health outcomes in rural settings.
**Barriers**			
Work life balance	The majority (85%) agreed that burnout and work overload is a barrier to acquiring AST with a mean score of 4.31 ± 0.79.	*“It was particularly challenging because I’m a single dad and I managed to get a spot in [regional center], but my kids were in [major city]. So that year alone, I drove almost 50,000 Kilometers between [major city] and [regional center], and on my 2 days off, I’ll finish at 5 or 6 or 7 p.m. then I will drive down to [major city] for 1 day. I will see my kids and family for 1 day and drive back that was quite challenging, but I just had to be resilient.” Fellow 7, Male.*	The results underscore the need for comprehensive support systems, flexible training structures, and strategic planning to address the unique challenges facing rural generalist trainees. These measures are essential to ensure the wellbeing of trainees and the success of the AST program.
Inadequate knowledge and recognition of AST	About 74% of the responding GPs perceived professional recognition and career development through AST as highly important.	*“And I think the other thing is. It wasn’t so bad in [regional center], but I know that there’s places where the cultural attitude toward general practice and rural GPs is less than positive because there is a metro centric sort of attitude in health which says that if you’re not in the big city, you’re a failure. So there’s, you know, [regional center’s not so bad because [major city] thinks [regional center] is the rural sticks and it’s not. It’s a big city.” DMS 3, Male.*	It is necessary to address metro-centric biases against rural generalists through education and branding. Changing perceptions, improving training environments, and providing strong support systems can significantly enhance the recruitment, retention, and wellbeing of rural GPs.

## Discussion

Building health workforce through additional skills is essential to promoting health accessibility and equity, especially for those in rural and remote Australia (24). AST empowers GPs to acquire additional skills to provide essential services in these areas (11, 13). This mixed-methods study explored the extrinsic and intrinsic factors influencing GPs choice of AST and their perceptions of the enablers and barriers to the AST program.

Quantitative findings revealed that GPs reported improved patient outcomes, increased patient satisfaction, and expansion of services offered as benefits of AST. However, significant barriers included funding challenges, burnout, workload, and low availability of mentors. Most GPs emphasized the need for policymakers and healthcare organizations to prioritize AST for GPs and healthcare providers in general and provide more opportunities to undertake the AST to support the delivery of specialized and essential services to their respective communities (15). The WHO (25) has also argued for increased visibility of training programs to enhance impact on delivery of effective health services. Intrinsic motivations for pursing AST included personal interest and passion, desire for professional growth and development, and a desire to improve patient care and outcomes. Qualitative findings highlighted enablers such as desire to work rurally (intrinsic motivation), meeting workforce needs, and support networks. Conversely, barriers included poor work-life balance, mismatch expectation from different stakeholders involved in the AST, and inadequate knowledge and recognition of AST.

Overall, the study findings reinforced that while GPs are willing to undertake AST, their main motivation is intrinsic, with financial compensation being less of a priority for participation (26). Although finances were not a primary motivation for participants pursuing AST, they faced significant financial challenges in practice. Participants reported issues such as loss of rank and salary, high housing costs, and hidden expenses related to travel. Previous studies have also argued that intrinsic motivation positively influences engagement in training and work (27, 28). However, work life balance remains a major challenge affecting learning, wellbeing during advanced training (29) and long term satisfaction (30, 31). Previous surveys have also indicated that 60% of physicians and 58% of trainees are dissatisfied with their work-life balance (32, 33). Poor work-life balance often leads to reduced work hours, practice changes, and exiting the medical field, negatively impacting the affordability and availability of care. Improving work-life balance for all trainees is crucial and requires organizational changes, such as flexible arrangements and adaptability (29). Additionally, fostering strong social support networks could help mitigate training pressures (29).

The findings also showed that inadequate knowledge and recognition of AST was a barrier. Previous studies have also shown a similar challenge with recognition of AST (2, 14). Specifically, these studies (2, 14) have all found that the level of recognition was limited in the general healthcare system although communities, GPs and some peers appreciate the value of AST. The findings also showed that the top five ASTs pursued were anesthetics, emergency medicine, and obstetrics and gynecology, whilst the top five additional or extended skill sets surveyed GPs reported they use daily were chronic disease management, mental health, internal medicine, pediatrics, and adolescent and youth health. It is important to add that respondents might have provided the skills they use daily in their GP role instead of the additional skills. Nonetheless, this may imply skills mismatch at the community level as discussed in a previous study (34). Mason et al. (34) reported that participants emphasized the need to align AST specialty choices with community needs prior to starting training. This approach helps ensure that the skills gained address existing gaps and that there are opportunities for the GP to return and practice within the community. This also points to the fact that skills other than procedural skills are also more required in rural and remote settings (3) to manage their disease and health conditions in general. This suggests that funding allocation could also prioritize widely used skills, particularly in chronic diseases and mental health, which are prevalent in rural and remote areas (6, 35). The need to also pay attention to mental health and chronic diseases reflects the disease burden globally and specifically in Australia (35). Hence, the skills needed should align with these prevalent health issues in the respective communities (12).

### Implications for policy and practice

The study findings have implications for policy and practice. In terms of ensuring that the AST meet the community needs, it is essential that specific skills needed in rural and remote communities in which RGs work are promoted. It also requires that the trainee receive timely advise from career development practitioners to inform their selection of AST (14). This sequential approach could address the current mismatch in the expectations where AST is completed before GPs begin to establish their practice within a community. Second, enhancement and promotion of utility of AST is needed. This will ensure that it is highly valued and recognized within the GPs space and among prospective RGs. Third, providing incentives such as scholarships and mentorship avenues are crucial for the success of AST program.

### Strengths and limitations

This study provides key insights into the enablers and barriers faced by GPs in pursuing AST. By employing a mixed methods approach, it integrates both quantitative and qualitative data to enrich the study’s findings. However, some limitations are worth discussing. First, this study included only the views of fellows and DMS. Second, the qualitative phase of the study did not cover all AST programs, hence the possibility of missing some perspectives. Although the study invited GPs across Australia, most participants, particularly, in the qualitative phase, were currently employed in Queensland, where pathways for rural generalist are available (34). Future studies should include more respondents from other states and territories in Australia. Additionally, there is a possibility of sampling bias, as probably only those who showed interest participated in the study. Moreover, there is possibility that sampling bias and social desirability biases of responses in both phases of the study are possible.

## Conclusion

To conclude, AST is a highly valued training program. Most GPs are intrinsically motivated to participate in the program. However, to ensure sustainability, wider recognition, better visibility, and better alignment with community needs need to be prioritized.

## Data Availability

The original contributions presented in this study are included in this article/supplementary material, further inquiries can be directed to the corresponding author.
